# Enhancing surgical occlusion setting in orthognathic surgery planning using mixed reality technology: a comparative study

**DOI:** 10.1007/s00784-024-05930-w

**Published:** 2024-09-24

**Authors:** Max Wilkat, Felix Schrader, Julia Trusch, Nadia Karnatz, Kathrin Becker, Leonardo Saigo, Majeed Rana

**Affiliations:** 1 Department of Oral and Plastic Maxillofacial Surgery, Heinrich Heine University Hospital Düsseldorf, Moorenstraße 5, Düsseldorf, 40225 Germany; 2 Department of Orthodontics and Dentofacial Orthopedics, Charité - Universitätsmedizin Berlin, corporate member of Freie Universität Berlin and Humboldt-Universität zu Berlin, Aßmannshauser Str. 4-6, Berlin, 14197 Germany; 3 Department of Oral and Maxillofacial Surgery, National Dental Centre Singapore, 5 Second Hospital Ave, Singapur, 168938 Singapore

**Keywords:** Virtual occlusion, Mixed reality, Orthognathic surgery, Intra-oral scanning, Computer-assisted planning, Surgical occlusion

## Abstract

**Objectives:**

Orthognathic surgery necessitates precise occlusal alignment during surgical planning, traditionally achieved through manual alignment of physical dental models as the recognized gold standard. This study aims to evaluate the efficacy of mixed reality technology in enhancing surgical occlusion setting compared to traditional physical alignment and an established virtual method, addressing the research question: Can mixed reality technology improve the accuracy and efficiency of occlusion setting in orthognathic surgery planning?

**Materials & methods:**

This experimental study compared the surgical occlusion settings of 30 orthognathic cases using three methods: a new virtual method with mixed reality technology, the traditional gold standard of physical alignment, and an established virtual occlusion method using the IPS Case Designer (KLS Martin SE & Co. KG, Tuttlingen, Germany).

**Results:**

Results indicated that surgical occlusions set with mixed reality technology were comparable to the conventional method in terms of maxillary movement and occlusal relationship. Differences observed were within the inter-observer variability of the gold standard. Both virtual methods tended to position the maxilla more anteriorly, resulting in fewer occlusal contacts. However, virtual occlusion demonstrated clinical applicability, achieving an average of 11 occlusal contacts with a bilaterally symmetrical distribution along the dental arch.

**Conclusions:**

The mixed reality environment provides an intuitive and flexible experience for setting surgical occlusion, eliminating the need for costly 3D-printed physical models or the automatic calculations required by other virtual occlusion methods, thereby offering maximum freedom.

**Clinical relevance:**

As a novel form of virtual occlusion, it presents a comprehensive tool that contributes to a timely and cost-effective full digital workflow of orthognathic surgery planning.

## Introduction

Skeletal discrepancies within the maxillo-mandibular complex frequently manifest as malocclusion and facial aesthetic impairments, necessitating a combined orthodontic and surgical intervention for correction [1]. Traditionally, this treatment involves a three-stage approach comprising pre-surgical orthodontic preparation, orthognathic surgery, and post-surgical orthodontic to achieve an optimal occlusion. In recent years, a surgery-first approach has gained popularity due to its potential for shorter overall treatment duration and earlier resolution of facial deformities [2, 3]. However, regardless of the treatment approach, achieving optimal surgical occlusion is paramount.

Surgical occlusion, defined as the occlusal relationship established during orthognathic surgery, plays a crucial role in treatment outcomes. It is typically determined during pre-operative planning and aims to produce a “treatable malocclusion”, which should be correctable during the post-surgical orthodontics phase to avoid the need for revision surgery [4]. The complexity of post-surgical orthodontic correction is usually higher for surgery-first protocol as surgical occlusion is less ideal compared to orthodontic-first cases [5].

While virtual surgical planning has become a new norm in orthognathic surgery [6–8], the setting of surgical occlusion continues to rely predominantly on manual alignment of physical models, a process that provides tactile feedback to the user. The models are often manufactured out of plaster as dental stone models. The physically set occlusion is then digitized using a 3D scanner to enter the virtual workflow of computer-assisted planning. With recent advancements in intra-oral scanning technology, 3D-printed resin models are more preferred to the stone models today. The resin models are more resistant to erosion and abrasion, and has proven to be more accurate [9]. However, 3D-printing is still expensive and time consuming.

Besides the mode of manual alignment in a physical environment, there exists the concept of virtual occlusion. Virtual occlusion can be defined as an occlusion that is achieved by virtually aligning upper and lower digital dental models in a computer. However, an inherent issue of virtual occlusion is the incidence of interference [10]. In a real-world environment, two colliding objects will never interfere due to their impenetrable surfaces, which will lead to a force-feedback during contact or collision. Digital objects, however, will simply interfere when moved into each other assuming a relative position that is impossible to recreate in the real world without damaging the objects. Therefore, a solid collision detection system is mandatory for virtual occlusion setting. The most natural way to indicate a collision seems to be haptic feedback. There are prototypes which have been developed to re-create this force-feedback created during conventional occlusion setting with physical models [11, 12]. The central component of this prototype utilizes the Geomagic haptic device, comprised of a gimbal-driven stylus capable of tracking 6 degrees of freedom (DoF) movements and providing 3 DoF force feedback. Leveraging impulse-based dynamics, the occurrence of impulsive and contact forces during dental model collisions is computed and presented to the user’s sensory perception. Observations reveal that the prototype enables virtual occlusion setting with relatively minor deviations compared to the conventional gold standard of physical dental plaster cast occlusion setting [11]. However, notable limitations include the observed high latencies for high-resolution models, attributed to increased computational demands for concurrent collision detection and force simulation, thus impeding clinical applicability to date.

A more commonly adopted method involves visually driven modes of virtual occlusion, facilitated by tools such as occlusogram (also called occlusionogram). An occlusogram utilizes distance maps to illustrate the minimal distances between the surfaces of the upper and lower dentition. Additionally, advancements include automated or semi-automated computations to achieve a new position with desired occlusal features. One of the most prevalent techniques is the spring approach, first described by Nadjmi [13]. With this approach, three pairs of corresponding dental landmarks are first defined. Subsequently, a simulation is conducted, wherein a spring is stretched between each corresponding pair, calculating a new position of the upper model to approximate the pairs as closely as possible while ensuring no collision between the two models. The resulting occlusion can be assessed by the observer, with further adjustments of the springs and subsequent repetition of the simulation if necessary. This method has been incorporated into commercially available software packages like IPS Case Designer (KLS Martin SE & Co. KG, Tuttlingen, Germany). Its application has been investigated and endorsed for clinical use by some authors [14–16].

This virtual occlusion approach, like all others, heavily relies on visual approval, similar to the conventional method. However, visual assessment in virtual occlusion is hindered by the limitation of 2D computer monitor displays, resulting in a non-intuitive user experience that requires a steep learning curve.

Recent advancements in mixed reality technology offer a solution by providing fully immersive 3D displays of any desired data. Utilizing a head-mounted display with externally projected cameras and motion sensors, the device analyzes and interprets the real-world environment, projecting the data as an integrated object that mimics a physical object. This allows for intuitive inspection and interaction with virtual surgical planning data using a 6 degrees of freedom (6 DoF) motion controller.

We recently introduced a fully functional tool for virtual occlusion setting in a true 3D environment, utilizing the immersive capabilities provided by advanced mixed reality technology [17]. The key features include superior visualization of dental models, offering advantages such as scaling to enhance visibility of even small details on occlusal surfaces, and the ability to generate on-the-fly section views by navigating through the virtual models in 3D space by simple locomotion of the user. Occlusion setting is further enhanced by real-time collision detection, facilitated by algorithm optimization such as AABB tree build and server-based computational resources. Contact or collision detection can be conveyed through visual cues and haptic feedback on the controller. Additionally, detailed information can be extracted from the occlusogram.

The aim of the present study was to evaluate whether virtual occlusion enhanced by the new mixed reality tool is capable of creating a clinically desirable occlusion. Three different methods of setting surgical occlusion were compared in this experimental study setup: (1) conventional method by manual adjustment of resin casts as the gold standard, (2) mixed reality method as the newly developed mode of surgical occlusion setting, (3) digital method which comprises a validated method of virtual occlusion setting [14–16]. This study is the first study to the best of our knowledge that evaluates a tool for virtual occlusion setting in orthognathic surgery planning based on mixed reality technology in a true 3D environment.

## Materials & methods

### Subjects

This experimental study was approved by the Ethics Committee of the Heinrich Heine University Düsseldorf (study number: 2022–2229). The archived data of completed orthognathic surgery cases between 2018 and 2022 was used for this experimental study. Eligible patients who met the inclusion criteria were identified from the records of the Department for Oral & Plastic Maxillofacial Surgery at the Herinrich Heine University Hospital Düsseldorf. Inclusion criteria were as follows:

- Patients aged 18 years and above at the time of surgery, having legally consented to retrospective pseudonymized use of their data for research purposes.
- Angle class II or III malocclusion.
- Orthodontic-first surgical protocol.
- Six or more teeth per quadrant at the time of surgery without any supernumerary teeth.
- Presence of a complete and undamaged set of pre-surgical dental stone models and occlusal registration record.
- Presence of a complete DICOM data set of the pre-surgical cone-beam computed tomography (CBCT) scan.

Of all the identified cases, a total of 30 cases were randomly sampled to achieve an equal distribution of 15 cases of Angle class II and 15 cases of Angle class III malocclusion.

### Methods

Two observers (M.W. and L.S.) performed the occlusion setting for each of the three methods:

- M1 - Conventional method using 3D-printed resin models as gold standard
- M2 - Mixed reality method using Magic Leap One (Magic Leap Inc, Florida, USA) device and Brainlab Elements (Brainlab AG, Munich, Germany) software
- M3 - Digital method using the occlusion wizard of IPS^®^ CaseDesigner (KLS Martin SE & Co. KG, Tuttlingen, Germany).

**Fig. 1:**
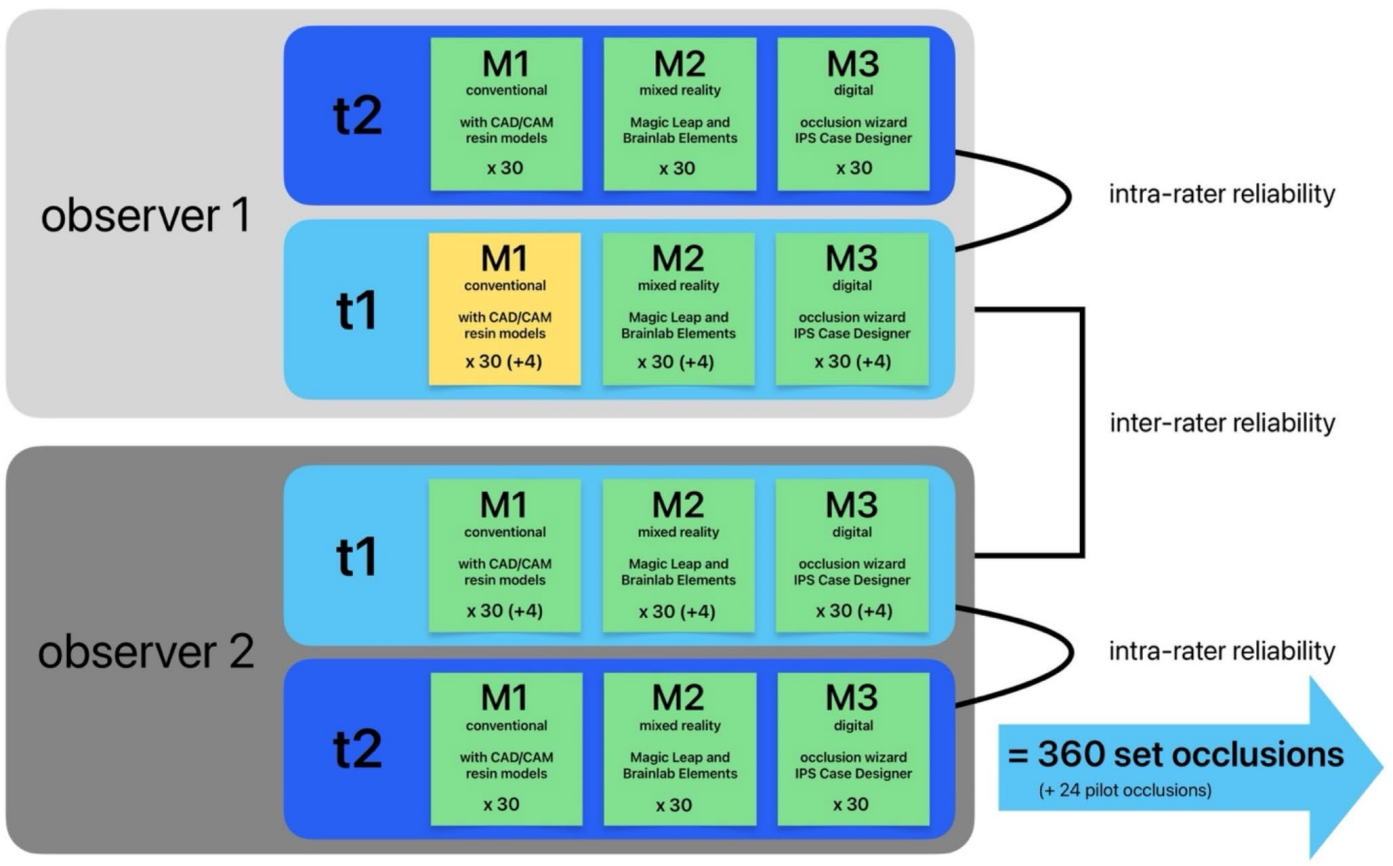
Study design. Two observers performed the experimental occlusion setting for each of the three methods M1, M2 and M3 independently from each other at two time points t1 and t2 which allowed evaluation of intra- and inter-observer reliability

These were done independently at two time points (t1 and t2) which were at least 4 weeks apart. The 30 cases were randomly shuffled at each time point to reduce bias. Four separate cases were used as “pilot cases” to familiarize the observers with the different methods of setting the surgical occlusion which were only performed once at t1. These pilot cases were not included for evaluation apart from assessing the different learning curves between the observers and methods. This study setup produced 360 surgical occlusion which were evaluated for intra- and inter-observer reliability. The study design is summarized in Fig. 1.

### Preparation of dental models

Upper and lower plaster models were surface-scanned using model surface scanner Zirkonzahn S600 (Zirkonzahn GmbH, Gais, Italy). The pre-operative occlusion was also scanned using the same method. Subsequently, digital pedestalling onto an orthodontic model base [18] was done using Autodesk Meshmixer (v3.5, Autodesk Research, San Francisco, CA, USA). This resulted in watertight STL files of the upper and lower dentitions. These STL files served as the common starting point for each of the three methods of surgical occlusion setting.

1. M1 - Conventional method: The digital models were made hollow with the addition of 4 drainage holes using Autodesk Meshmixer (v3.5, Autodesk Research, San Francisco, CA, USA). The STL files for 3D printing were imported into PreForm software (version 3.27.1, Formlabs Inc., Somerville, MA, USA), choosing dental model resin V3 (Formlabs Inc., Somerville, MA, USA) and a slice thickness of 0.05 mm. Support structures were calculated automatically and manually edited to remove those near the occlusal surfaces of the teeth. The final print job was uploaded to SLA printer Form 2, Formlabs (Formlabs Inc., Somerville, MA, USA). After printing was finished, the dental resin models were post-processed according to the manufacturer’s protocol (Formlabs Inc., Somerville, MA, USA). Using the Form Wash, the model was washed in 99% isopropyl alcohol for 20 min, air-dried for 30 min, and cured in the Form Cure at 60 °C for 30 min. Further post-processing included the removal of the support structures. The finished dental resin models were ready for conventional surgical occlusion setting by the observers as described below.
2. M2 – Mixed reality method: A detailed description of the hardware and software components has been published previously [17]. The STL files of the upper and lower dental models were loaded into the Brainlab Elements software (Brainlab Elements Viewer 5.3, Brainlab AG, Munich, Germany). Once a common wifi connection between the laptop running the software and the head mounted display Magic Leap One (Magic Leap Inc, Florida, USA) was established, the observer scanned the designated QR-code from the laptop screen to enter the planning session. Hence, the virtual occlusion setting with mixed reality started as described below.
3. M3 - Digital method: Virtual surgical occlusion setting was carried out using the orthognathic planning software IPS^®^ CaseDesigner (KLS Martin SE & Co. KG, Tuttlingen, Germany). Once the data was uploaded into the software, the occlusion wizard function could be opened for digital surgical occlusion setting as described below.

### Setting surgical occlusion

The process of setting the surgical occlusion was performed by two maxillofacial surgeons (M.W. and L.S.) experienced in orthognathic surgery independently at two time points. The second time point took place at least four weeks after the first to prevent memory bias. Moreover, the order of the models was randomly shuffled for each session.

1. M1 - Conventional method: The printed upper and lower dental models were put together in the surgical occlusion using the articulator clamp of model surface scanner Zirkonzahn S600 (Zirkonzahn GmbH, Gais, Italy). After the final surgical occlusion was approved, occlusion was scanned from the vestibular surface using the same scanner to create an STL file.
2. M2 -Mixed reality method: A detailed description of the process of setting surgical occlusion in the newly developed mixed reality environment has been described previously [17, 18]. In summary, after scaling the models to the desired size and putting the occlusion plane slightly beneath eye-level of the observer for convenient inspection, the rotation center was positioned at the lower dental midline. Initial alignment could be done by grabbing the upper model with the trigger button and moving it free-hand to the desired position. Ideal dental midline, overjet and overbite (translational movements) were first determined using touch gestures on the hand controller’s touch pad. Roll, pitch and yaw corrections (rotational movements) were then done using the swipe gestures. Fine adjustments of the occlusion could be achieved using the movement table interface, approximating the models just before an intersection was detected by the real-time collision detection system. The whole process was enhanced by superior visualization of the mixed reality environment, allowing free locomotion of the observer around and through the models leading to on-the-fly section views. Moreover, occlusal contacts could be reviewed in the occlusogram display. Once the final surgical occlusion was confirmed, an STL file was exported by a click of a button.
3. M3 - Digital method: The recommended steps of the occlusion wizard in IPS^®^ CaseDesigner (KLS Martin SE & Co. KG, Tuttlingen, Germany) were followed. First, three pairs of corresponding dental landmarks were defined (palatal proximal contact of upper first incisals to lower incisal point, mesio-palatal cusp of the upper right and left first molar to central fossa of the lower right and left first molar). Virtual springs were then created between the pairs to simulate a new position of the upper model which approximated the two points of the three corresponding pairs as close as possible without collision. The resulting occlusion was then reviewed. If necessary, further adjustments could be made by either moving the attachment point of the existing springs or adding further springs. Apart from or in combination with the spring approach, free translational and rotational movement of the upper model following the x-, y- and z-axis could be performed as well. Moreover, occlusal contacts could be reviewed in the occlusogram display.

### Evaluation of surgical occlusion

#### Data pre-processing for evaluation

Evaluation of occlusion was carried out using IPS^®^ CaseDesigner (KLS Martin SE & Co. KG, Tuttlingen, Germany). For each subject, DICOM data of the pre-surgical CBCT scan was loaded into the software. The virtual head was aligned according to the Frankfort horizontal and mid-sagittal plane. STL files of the upper and lower dentition were loaded into the software and automatically aligned to the DICOM data using a voxel-based matching algorithm. The matching result had to be approved by the observer. Cephalometric analysis was conducted indicating six dental landmarks onto the upper and lower jaw namely: incisal points (UI: upper incisal, LI: lower incisal) and mesio-buccal cusp tips of the first upper and lower molar (UMcusp(r): upper right molar, UMcusp(l): upper left molar, LMcusp(r): lower right molar, LMcusp(l): lower left molar). Le Fort I osteotomy lines were then outlined by the observer.

**Fig. 2:**
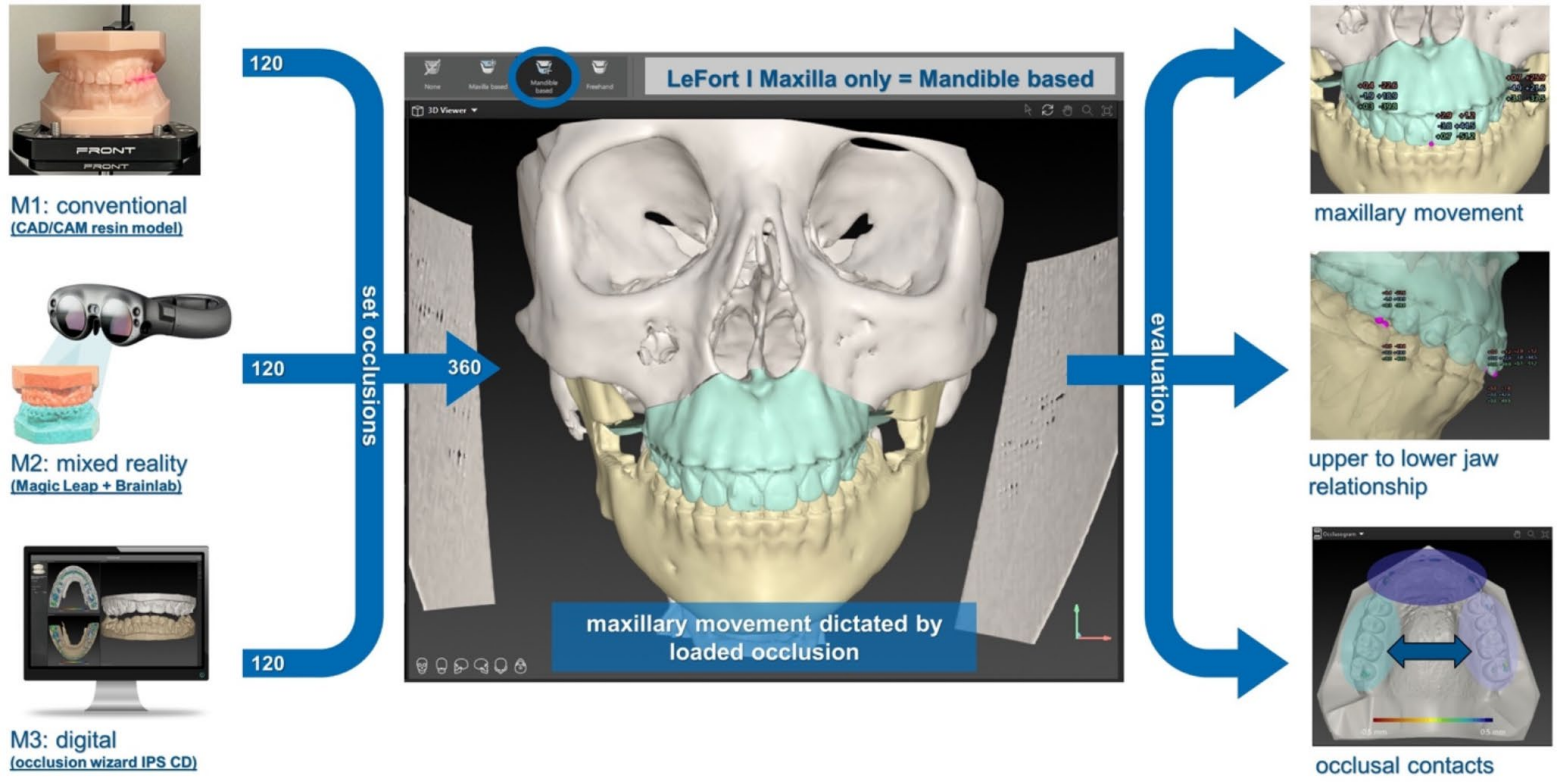
Summary of experimental setup and evaluation

The file was copied twelve times resulting in an individual file for each method, each observer and each time point. The final surgical occlusions from the experimental setup were loaded into the appropriate planning file following a mandible-based approach, which led to a maxilla-only movement. Thus, every case was virtually planned as a LeFort I osteotomy with the movement of the maxilla being only dictated by the set surgical occlusion. Using this, maxillary movement, upper to lower jaw relationship and occlusal contacts were evaluated. This process is summarized in Fig. 2.

#### Maxillary movements

**Fig. 3:**
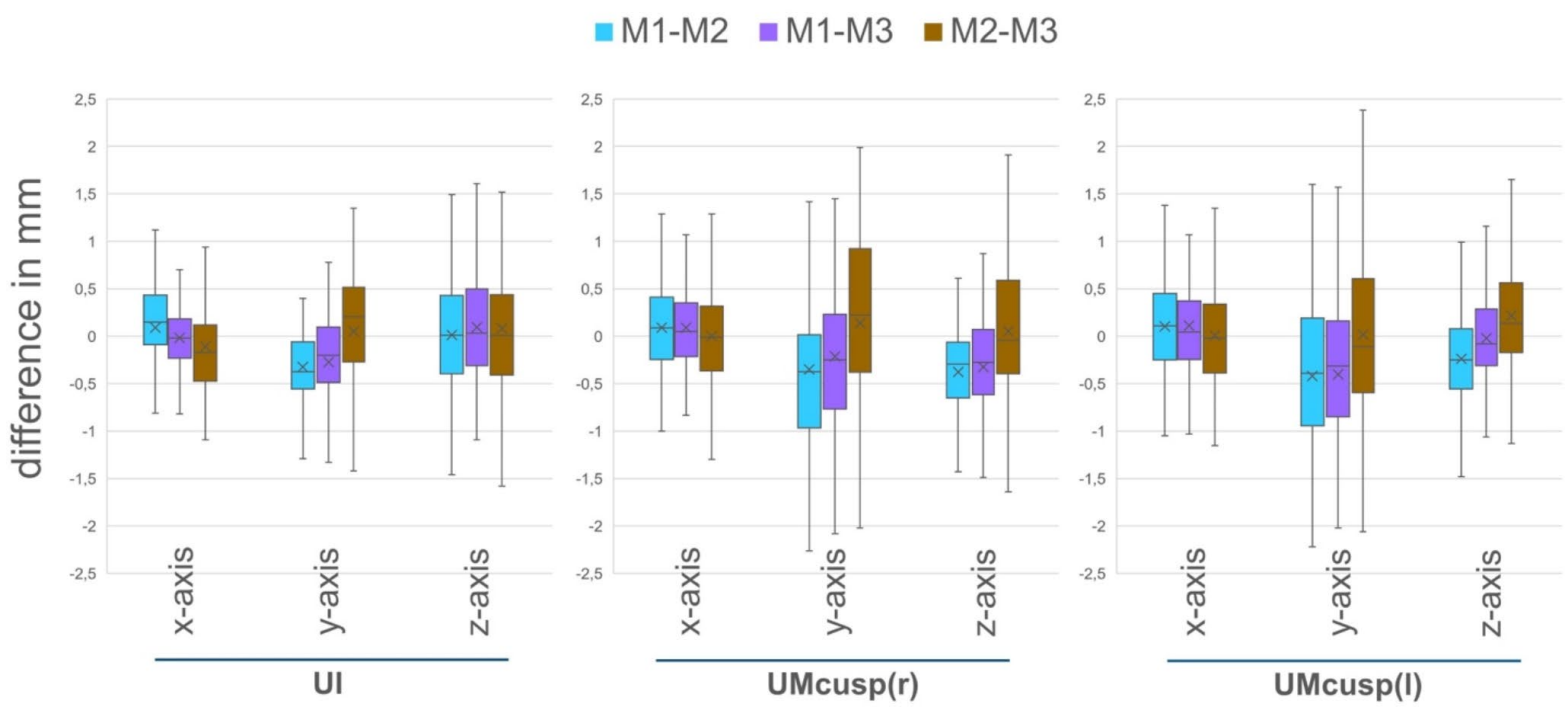
Boxplot of differences at the three coordinates: upper inicisal (UI), upper first molar cusp right (UMcusp(R)) and left (UMcusp(l)). Boxplots showing the minimum, 25-percentile, median, 75-percentile and maximum as well as the mean (marked by x) of the differences between the examined methods of surgical occlusion setting (M1-M2: blue, M1-M3: purple, M2-M3: brown). Differences are shown for each axis (x, y and z) for each of the three coordinates

**Fig. 4:**
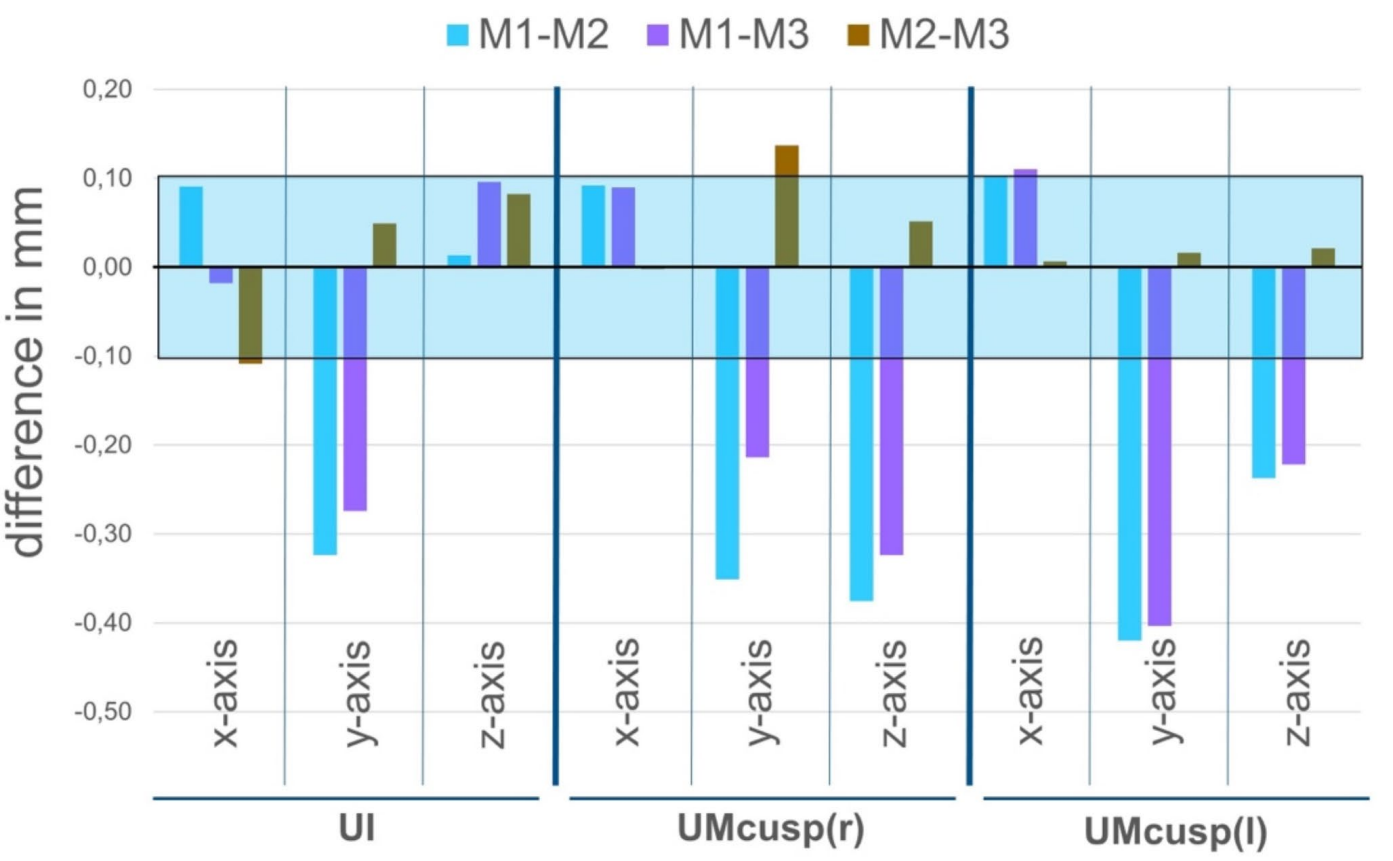
Mean differences at the three coordinates: upper inicisal (UI), upper first molar cusp right (UMcusp(R)) and left (UMcusp(l)). The mean of the differences between the examined methods of surgical occlusion setting (M1-M2: blue, M1-M3: purple, M2-M3: brown) are shown (compare Fig. 3). Differences are shown for each axis (x, y and z) for each of the three coordinates UI, UMcusp(r) and UMcusp(l). Marking of the light blue area with minimal differences of ± 0.1 reveal that the bigger differences are especially seen along y- and z-axis

**Fig. 5:**
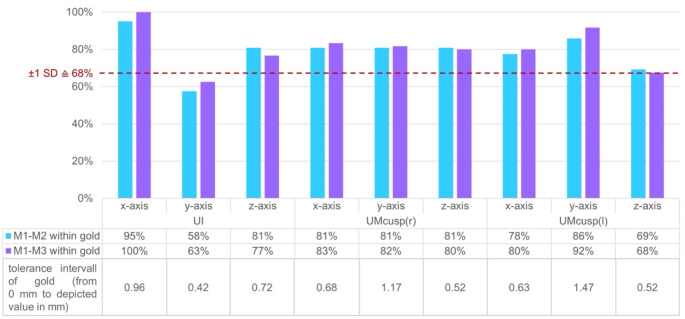
Percentage of values of M2 and M3 within the tolerance interval of the gold standard Graph shows the percentage of values for the difference of M1 to M2 and M1 to M3 which fall into the specified gold standard defined as the mean difference ± 1 SD between observer 1 and observer 2 for the conventional method M1. The 68-percentile line is marked in red to indicate the minimum limit which should be reached as it corresponds to the value of the mean difference ± 1 SD

The movement of the maxilla was assessed via the delta between the pre-op and planned coordinates of the following three dental landmarks: upper incisal point, mesio-buccal cusp tips of the upper right and left first molar. Data was analyzed for each axis: right (-) and left (+) along x-axis, down (-) and up (+) along y-axis, back (-) and forward (+) along z-axis. These values were calculated for each method. When comparing the methods, the difference between two methods was calculated showing the over- or undercorrection of the one method compared to the other method (Figs. 3, 4 and 5).

#### Upper to lower jaw relationship

**Fig. 6:**
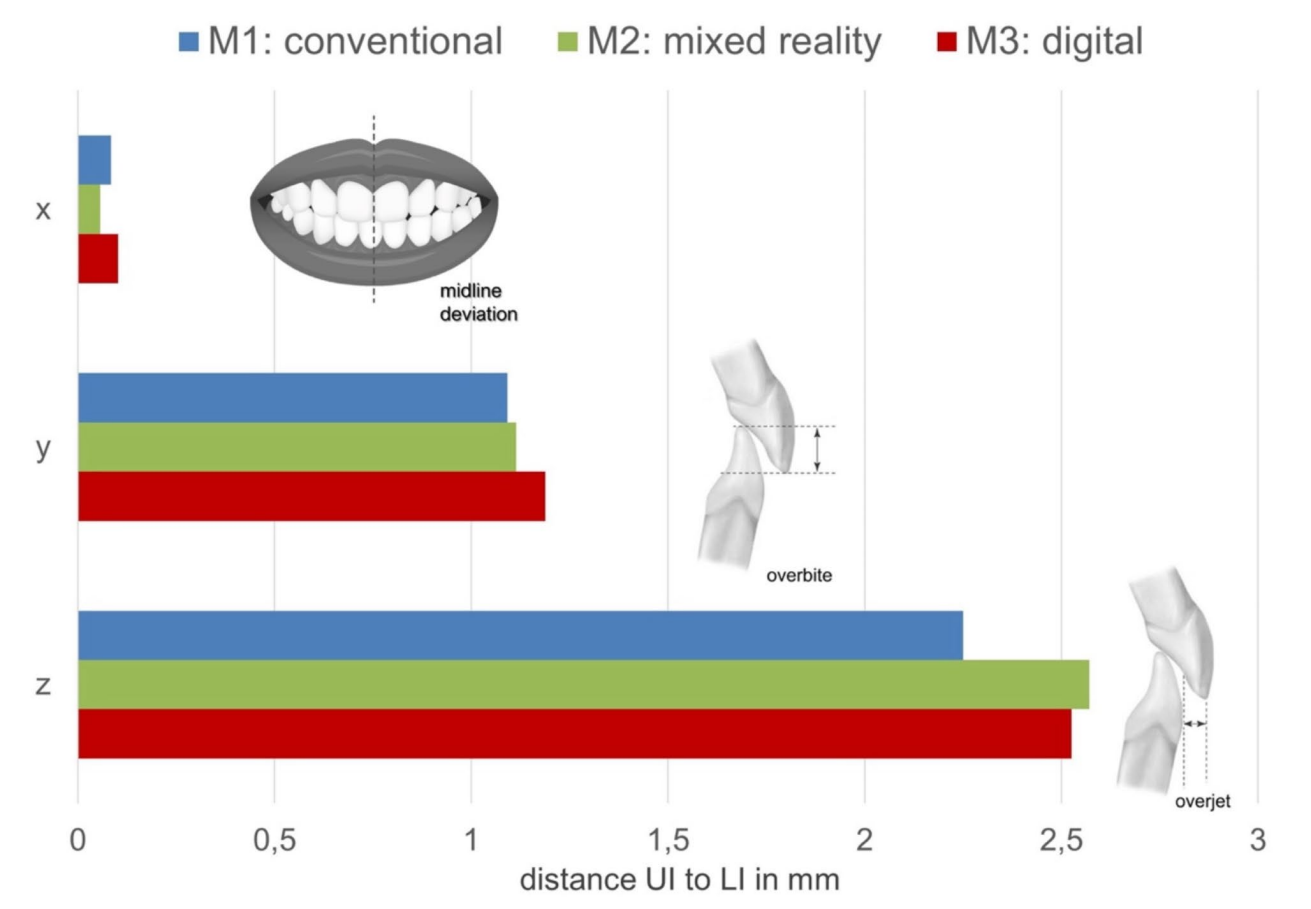
Absolute mean values for midline deviation, overbite and overjet among 3 methods. The delta between the upper and lower incisal corresponds to the midline deviation (x-axis), overbite (y-axis and overjet (z-axis) of the set surgical occlusion. Data is shown for each of the methods M1 (blue), M2 (green) and M3 (red)

**Fig. 7:**
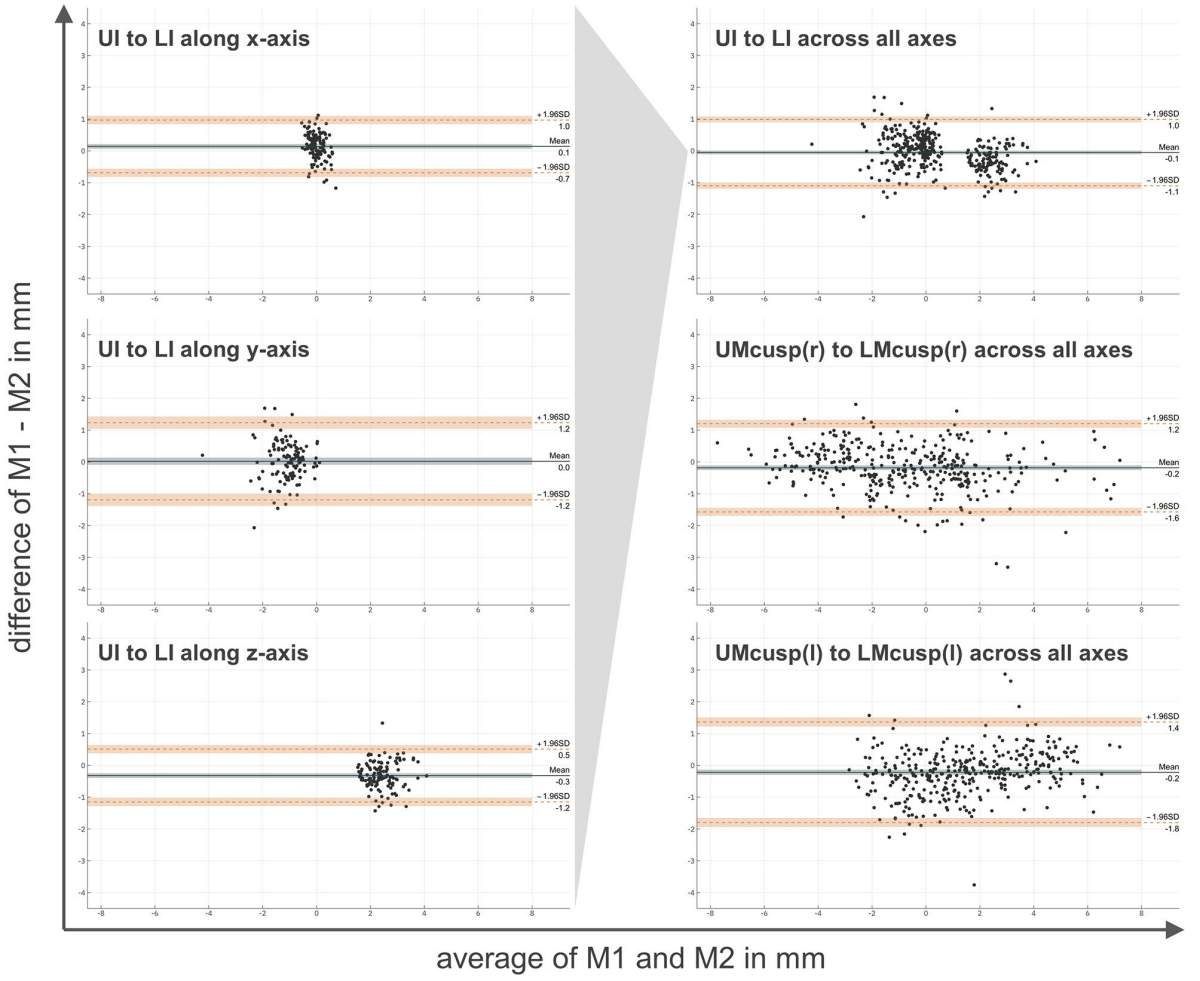
Bland-Altmann plots for the incisal relationship (presented separately for each axis on the left side, summarized across all axes on the upper right side) as well as the molar relationships right and left (summarized across all axes on the middle and lower right side) comparing the conventional method M1 with the mixed reality method M2. The x-axis of the graph shows the average of the two methods for each data point while the y-axis shows the difference between M1 and M2

For each method, the relationship between the upper to lower dentition was analyzed by calculating the differences between the coordinates of the upper incisal point (UI) to lower incisal point (LI) along each axis. Midline deviation (x-axis), overbite (y-axis) and overjet (z-axis) were assessed and compared between the different methods (Figs. 6 and 7). Similar analysis could be made for the posterior segments on both sides by calculating the differences between the coordinates of the mesio-buccal cusp tips of the upper first molar and the the lower first molar on each side (Fig. 7).

#### Occlusal contacts

**Fig. 8:**
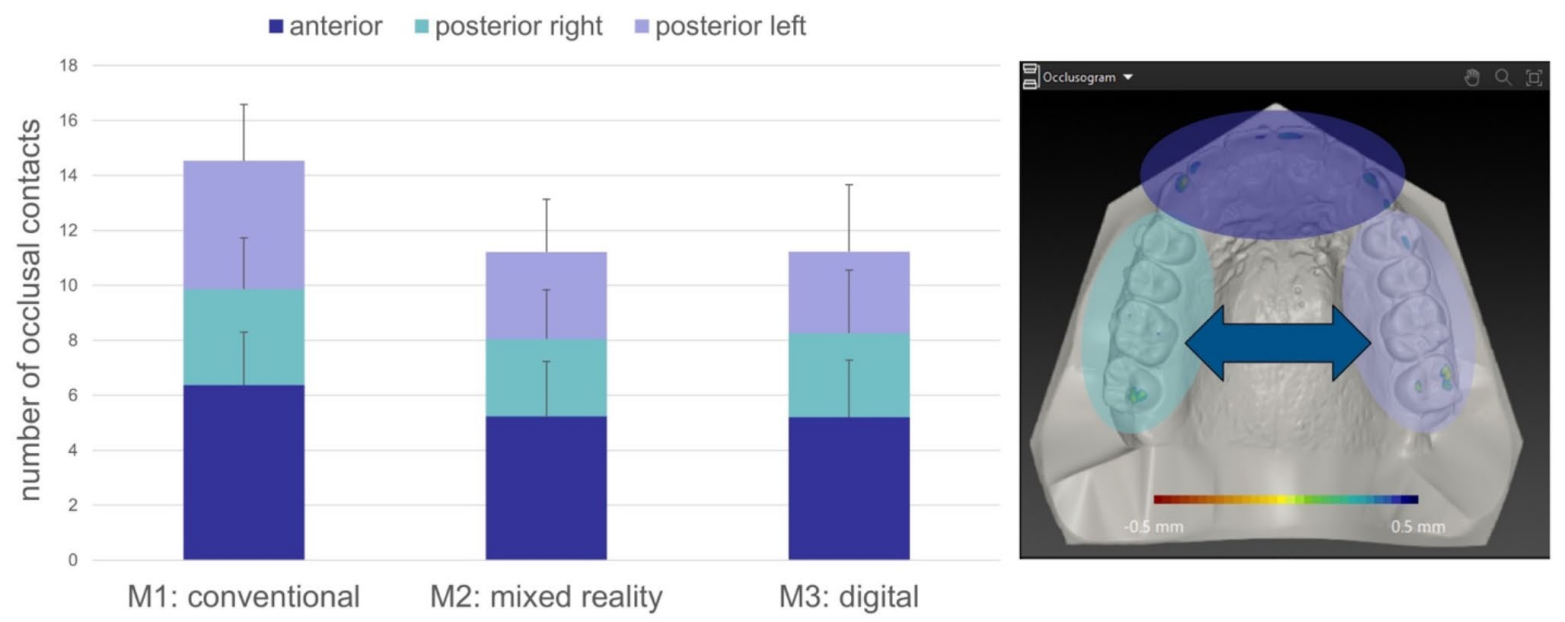
Number and distribution of occlusal contacts on the upper jaw. Occlusal contacts defined by a distance from 0 mm to a maximum of + 0.5 mm in the occlusogramm are displayed across the dental arch of the upper jaw. Mean number of occlusal contacts for each of the three methods M1, M2 and M3 are displayed divided by the dental arch segments: anterior (dark blue), posterior right (turquoise) and posterior left (purple)

For each method, the amount and distribution of occlusal contacts were evaluated. Only the upper dentition occlusogramm was assessed. The upper dental arch was divided into three segments: anterior segment (right to left canine), right posterior segment (right first premolar to second molar) and left posterior segment (left first premolar to second molar). All occlusal contacts were counted for each segment. One occlusal contact was defined by a coherent area of 0.5 mm or less distance to the lower dental arch surface (Fig. 8).

### Time analysis

**Fig. 9:**
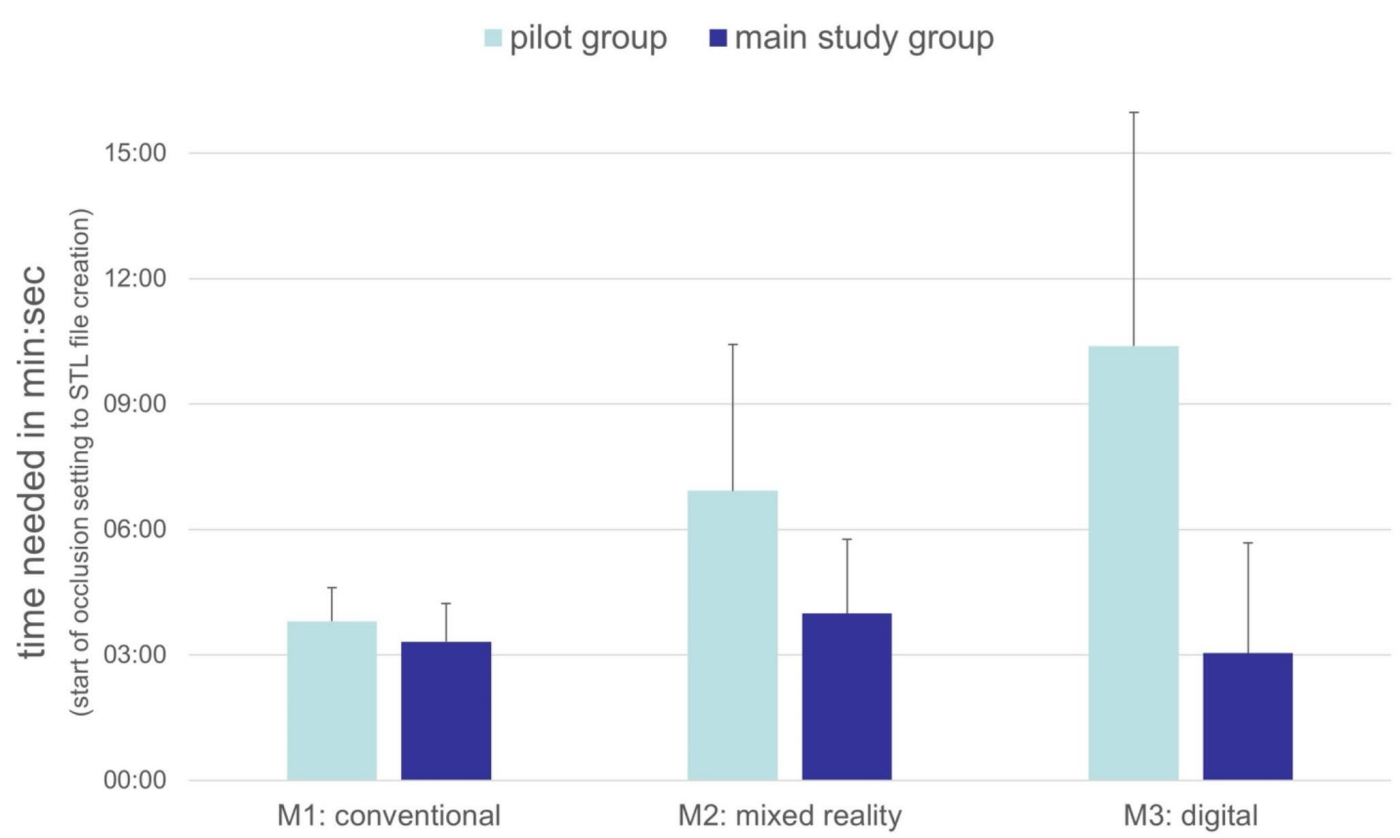
Comparison of mean time taken among 3 methods. Times in min: sec were taken for the 4 cases of the pilot series, which served for the observer to get familiar with the method, as well as for the 30 cases of the main study. Displayed are the mean and standard deviation of the time needed for each method. Pilot series are displayed in turquoise and the main study cases are displayed in dark blue

Time taken for each method was recorded from start of surgical occlusion setting until the final STL file was created. Average times were calculated for the four pilot cases and the 30 experimental cases (Fig 9).

### Statistics

All data collection and storage were carried out using Excel spreadsheets (Excel 14.0, Microsoft Corporation, Washington, USA). The statistical evaluations were carried out with the software IBM SPSS Statistics (IBM Corp. Released 2023. IBM SPSS Statistics for Macintosh, Version 29.0.1. Armonk. NY: IBM Coro).

Intra-rater and inter-rater reliability were assessed by calculating the ICCs (Intraclass Correlation Coefficients). The provided ICCs indicate the level of absolute agreement between different measurements. The values range from 0 to 1, where values between 0.75 and 0.90 are considered good, and values above 0.90 are considered very good agreement/reliability. For the intra-rater reliability, all values at t1 and t2 were compared separately for each method (M1 to M3) and each coordinate (UI, UMcusp(r) and UMcusp(l)) along each axis (x, y and z). For the inter-rater reliability, all values of observer 1 and observer 2 were compared with each other across all time points (t1 and t2) for each method (M1 to M3) and each coordinate (UI, UMcusp(r) and UMcusp(l)) along each axis (x, y and z).

For the upper to lower jaw relationship, a comparison of the conventional method M1 as the gold standard and the newly developed mixed reality method M2 was executed by calculating a Bland-Altmann plot (Fig. 7). The differences between M1 and M2 were plotted against their average. Moreover, the 95%-confidence interval was marked by indicating the ± 1.96 standard deviation (SD).

In order to record the statistical relationships between the groups in terms of maxillary movements and time assessment, they were checked for normal distribution using the Shapiro Wilk test and a graphical analysis using histograms and quantile-quantile plots. As normal data distribution was shown, differences were calculated with student’s t-test. Results with a p-value of less than 0.05 were considered significant.

## Results

### Intra- and inter-rater reliability

For intra-rater reliability, ICCs consistently showed very good results, with all values exceeding 0.90 except for two values for observer 2 and method 3 at the x-axis of UMcusp(r) and UMcusp(l) with an ICC of 0.88 and 0.87 respectively. The lowest value recorded was 0.94 for observer 1 and 0.87 for observer 2.

For inter-rater reliability, ICCs consistently showed very good results, with all values exceeding 0.90. The highest value recorded was 1.00, the lowest value recorded was 0.90.

### Maxillary movements

Overall differences between the conventional method M1 and the two virtual methods M2 and M3 revealed that differences along the x-axis were the smallest, especially for UI (Fig. 3), where values rarely exceeded ± 1 mm. For UI, differences between the methods were biggest along the z-axis reaching values of ± 1.5 mm. However, more than half of all differences (25- to 75-percentile) of UI did not exceed ± 0.5 mm for all of the three axes. For the coordinates of the posterior dental arch UMcusp(r) and UMcusp(l) values show a slightly bigger dispersion especially for y- and less for z-axis.

Mean differences between the different methods are shown in Fig. 4. For both the two virtual methods M2 and M3, there was a tendency of increased z-values in the anterior segment and a consequently increased y-value in the posterior segment which means that the maxilla was positioned further forward, and due to the anterior contact, the posterior region was more opened resulting in the observed values. However, mean differences along the y- and z-axis never exceeded − 0.5 mm.

To further investigate the clinical relevance of the deviations observed between the conventional method M1 and the two virtual methods M2 and M3, an acceptable range of variation for the differences between methods was defined around the absolute mean difference ± 1 SD based on the differences between observer 1 and 2 across both time points t1 and t2 for the conventional method M1, which served as the gold standard. For each coordinate point (UI, UMcusp(r), UMcusp(l)) and for each axis (x-, y-, and z-axis), the number of values obtained from methods M2 and M3 within the according calculated range of variation for the gold standard method M1 was determined. By definition, approximately 68% of values should fall within this gold standard interval if the data points were normally distributed, indicating that the spread of values from the other method matched that of the gold standard. As shown in Fig. 5, for all coordinates and axes of methods M2 and M3, more than 68% of all values fell within the specified interval of the gold standard except for the y-axis of the incisal point which was probably due to the fact that the gold standard interval for this coordinate was smallest with an interval of 0–0.42 mm. Since graphical analysis using histograms and Q-Q plots revealed a normal distribution for all values across all methods, it can be assumed that the spread of the two investigated virtual methods M2 and M3 is equivalent to that of the gold standard M1, and thus they can be considered equivalent in terms of maxillary movement.

### Upper to lower jaw relationship

Absolute mean values of the dental midline deviation (x-axis) are shown in Fig. 6. Method M2 showed the least midline deviation with a value of 0.06 mm followed by M1 with 0.08 mm and M3 with 0.1 mm. Values were equally distributed to both right and left sides for all methods as there was no systematic bias.

Along the y-axis, mean values of incisal overbite were + 1.08 mm for M1, + 1.11 for M2 and + 1.19 mm for M3. All methods produced a desirable overbite within 0–3.5 mm according to the index of orthodontic treatment need [19, 20]. None of the set surgical occlusions showed an anterior open bite.

In the z-axis, a bigger discrepancy was seen between M1 with a mean overjet of + 2.25 mm and the two virtual methods M2 and M3 with mean overjet values of + 2.57 mm and + 2.52 mm respectively All methods produced a desirable overjet within 0–3.5 mm according to the index of orthodontic treatment need [19, 20].

Bland-Altmann plots show the agreement between two methods by plotting the difference between them against their mean. Figure 7 shows Bland-Altmann plots separated by each axis (x, y, z) for the incisal point relationship and across all three axes for the incisal point relationship and molar relationships comparing the conventional method M1 with mixed reality method M2. As the means were close to 0 with values of -0.1 mm for the incisal point relationship and −0.2 mm for the molar relationship on both sides, there was little to no systematic bias between the two methods. Biggest systematic error was found for the z-axis at the incisal point with a mean difference of -0.3 mm confirming a tendency of a more anteriorly positioned maxilla with the mixed reality method compared to the conventional method. The level of agreement between the two methods was high for all of the three relationships as there were only very few outliers without a recognizable pattern and no funnel-shaped distribution of datapoints, indicative of a consistent difference between the measurements without a systematic error.

### Occlusal contacts

Mean number and distribution of occlusal contacts in the three groups are displayed in Fig. 8. While overall contacts were highest for M1 (14.5), both virtual methods M2 and M3 showed a comparable number (11.2). Differences between M1 and the two virtual methods were significant (*p* < 0.01), without significant differences between the two virtual methods M2 and M3 (*p* = 0.97). However, relative distribution of occlusal contacts across the dental arch were similar for all three methods with 44%, 47% and 46% in the anterior segment, 24%, 25% and 27% in the right posterior segment and 32%, 28% and 26% in the left posterior segment for M1, M2 and M3 respectively. Thus, for all three methods, we found stronger occlusal contacts in the anterior segment and a balanced occlusion between both sides in the posterior segments. In addition, interferences defined by negative values in the occlusogramm were not observed in any of the set surgical occlusion for all three methods.

### Time analysis

The mean time taken to set surgical occlusion in four pilot and 30 experimental cases is shown in Fig. 9. While there was no significant difference between pilot and experimental cases for method M1 with 03:48 min and 03:19 min respectively (*p* = 0.68), times differed significantly between pilot and experimental cases for methods M2 and M3 with 06:55 min to 03:59 min for M2 (*p* = 0.03) and 10:23 min to 03:03 min for M3 (*p* < 0.01). There was no significant difference in the mean time taken for the experimental cases between the three methods (*p* > 0.05).

## Discussion

There are three approaches to obtain a virtual occlusion – automatic, semi-automatic and manual [10]. The automatic approach involves a computer algorithm which calculates a favorable occlusion based on automated analysis of dental arch features thereby defining occlusal pairs to be approximated as close as possible without generating interference [21, 22]. The defined occlusion is set and cannot be altered by the user in this fully automatic approach. On the other hand, the semi-automatic approach allows users to define the corresponding dental landmarks and subsequently follows the same automatic calculation of maximal approximation of corresponding landmarks without collision [13]. Unlike the automatic method, this approach allows users to redefine the landmarks if the resulting occlusion is unsatisfactory.

However, certain malocclusion/malformations demand an individualized approach [23–27]. An example of such scenario is class II division 2 malocclusion with reduced anterior face height. Orthodontists may intentionally maintain the curve of Spee to plan for a tripod surgical occlusion to increase facial height while correcting the class II relationship. In cases with severely proclined upper incisors, larger amount of pitch rotation may be required, which may create a surgical occlusion with posterior open bite in need of post-surgical orthodontic extrusion if retrusion of the mandible and multi-segmental osteotomy are not desired. In addition, most surgery-first cases will not work with an automatic approach as the dental arch alignment is not yet aligned and decompensated. In these cases, the surgical occlusion must allow adequate space needed for post-surgical orthodontic correction. Conventional setup of surgical occlusion for the aforementioned cases usually involves securing the desired occlusion using wires and wax as the bite is typically unbalanced and unstable. This cannot be reproduced by the automatic or semi-automatic approach as this would require vectors for locking with a clear definition of the distance and direction of locking.

Manual setting of virtual occlusion comprises of free movement of one jaw, typically the maxilla, by translation and rotation until a favorable surgical occlusion is achieved [10]. The advantage of this approach is the unrestricted freedom in occlusion adjustment, allowing for the management of the case scenarios described above. Without a haptic simulation framework [11], this method depends heavily on visual cues to determine occlusal contacts as stated by many authors [10, 13]. Such cues can be given by an occlusogram as a distance map indicating areas of occlusal contact or interference. Liu et al. tried to add further information to users by introducing a distance projection view through vector visualization [28]. For this, the user has to manually identify 37 pairs of dental landmarks between which vectors are projected onto the occlusal plane to demonstrate sagittal and transverse deviations. The authors found a median accuracy of 1 mm for their method compared to gold standard. However, their validation study was based on dentitions with normal occlusions. Nonetheless, the process of reaching a surgical occlusion via manual setting can be laborious and time-consuming as dental interferences are hard to identify on a two-dimensional (2D) computer screen.

The newly developed mixed reality method allows more superior visualization over the other methods of virtual occlusion. The 3D-hologram projection allows free locomotion of the user around the virtual dental models, mimicking the process of inspection during conventional method using physical dental models. In addition, virtual models can be scaled to serve various objectives. For example, they can be minimized for users to appreciate the midline and occlusal cant. Similarly, they can be maximized when users are scrutinizing occlusal interferences. Moreover, it adds the possibility of sectional views by simply stepping through the models which is not possible in a real-world environment with physical models and is less intuitively feasible on a 2D computer screen. These sectional views allow a detailed evaluation of overbite, overjet and bilaterally balanced contacts. Although the mixed reality method essentially uses the concept of manual approach to virtual occlusion setting without any automated computer algorithm similar to the protocol of Ho et al. [29] and Seo et al. [30], the superior visualization and additional features help enable a user-friendly experience with true 3D interaction.

The results of this study showed that the mixed reality method (M2) produced surgical occlusions that were comparable to the conventional method (M1), which was regarded as the gold standard. Compared to the digital method (M3) of virtual occlusion using the occlusion wizard of IPS^®^ CaseDesigner (KLS Martin SE & Co. KG, Tuttlingen, Germany), it could also achieve highly comparable results. Authors have previously studied the digital method in a similar experimental set-up as the current study, comparing it to the gold standard of conventional occlusion setting [14–16].

Baan et al. [14] evaluated surgical occlusion methods in 17 Class II patients prepared for bimaxillary surgery. They compared conventional occlusion, based on alginate impressions and plaster casts, to virtual occlusion using digitized casts and the IPS CaseDesigner. The study found that the smallest discrepancies were in cranial/caudal movement, while the largest were in yaw rotation, concluding that the virtual tool was clinically accurate. Awad et al. [15] compared 25 orthognathic surgery cases, using either conventional occlusion with plaster casts and CBCT scans or virtual occlusion with direct intraoral scanning. Despite different dentition capture methods, they found no significant differences in mandibular position post-surgery. However, only translational movements were reported, with the greatest discrepancies in cranial/caudal and back/front axes. They concluded virtual occlusion was applicable, with most discrepancies within ± 2 mm, acceptable for orthognathic procedures. Sabev et al. [16] studied 23 Class II and III patients, comparing conventional occlusion to digital occlusion using IPS CaseDesigner. They found the digital protocol, involving the spring approach followed by manual adjustments, had accuracy within ± 2 mm/°, with the largest deviations in anterior/posterior translation and yaw rotation. They concluded that both methods were comparable in accuracy.

Considering the above-mentioned studies, the current study had a comparable sample size of 30 patients (15 class II and 15 class III). The utilization of 3D-printouts for the conventional method led to very comparable results as all investigated methods were based on the same virtual impression by model scanning of the archived dental models. Moreover, usage of resin models instead of plaster prevents abrasion, which might have led to a limited comparability after repeated passes. Sabev et al. also used 3D-printouts for the conventional method, although printouts were based on direct intraoral scanning.

The method of analysis in the above-mentioned studies was achieved by mesh-based alignment of the maxillary or mandibular STL files using various software. We used the automatic voxel-based alignment for the upper and lower jaw in their original position, matched to the patient’s CBCT scan using IPS^®^ CaseDesigner (KLS Martin SE & Co. KG, Tuttlingen, Germany) which led to precisely the same starting position for each method as voxel-based matching is recognized as the mode of matching with the lowest matching bias. Maxillary movement was calculated using mesh-based matching of the surgical occlusion STL file.

In the current study, intra- and inter-rater reliabilities showed good to very good results, comparable to the above-mentioned studies. While most studies evaluated the movement of the maxilla or mandible along translation and rotation, we evaluated the maxillary movements by three coordinates. To define an interval of tolerance for the observed deviations between the different methods of occlusal setting, we used the mean absolute difference ± 1 SD between the two observers of the conventional method M1 as the gold standard, as the naturally occuring deviation between set occlusions of two experienced maxillofacial surgeons is also being accepted in clinical practice. Baan et al. similarly assessed the difference between the gold standard and the virtual group by comparing it to the intra-observer variability for the gold standard which was found 0.20 mm smaller than the variation between the different methods of occlusal setting [14]. Awad et al. as well as Sabev et al. referred to the usually achievable surgical accuracy of < 2 mm/° for the definition of a tolerance interval [15, 16]. However, if accuracy on the level of surgical occlusion is allowed to deviate from the conventional gold standard by such an amount, it might add up with the deviation of the accuracy of the surgical plan transfer leading to an overall deviation of up to ± 4 mm/° in a worst-case scenario which can hardly be clinically acceptable.

Assessing the movement of one jaw does not provide insight into specific occlusal features, even if deviations from the gold standard were minimal. Therefore, the current study additionally evaluated certain aspects of occlusion, namely the inter-jaw relationship with overbite, overjet and midline deviation, as well as the number and distribution of occlusal contacts. Although a lack of these features is often cited as a counterargument against virtual occlusion, previous studies on virtual occlusion did not evaluate these features at all.

We observed a slight increase in the overjet with the two virtual methods compared to the conventional method while the overbite in the anterior region remained unchanged. This consequently also led to a slightly more open bite in the posterior segment, as indicated by the increased values along the y-axis in the posterior segment. In the virtual world, the maxilla tends to shift forward causing the maxilla to tilt slightly over the anterior contact surfaces opening up in the posterior region. This deviation is likely found due to two reasons: the missing or reduced haptic feedback and the less stringent control mechanism along the z-axis (back/front), as noted by Baan et al. and Sabev et al. [14, 16]. This consequently led to a reduction in occlusal contacts while maintaining bilateral symmetrical distribution. Lo et al. investigated if skeletal stability was dependent on the surgical occlusion in Angle Class III cases following a surgery-first protocol [31]. Skeletal stability was defined as the change of maxillary and mandibular positions in CBCT scans taken directly post-surgical and after finishing post-surgical orthodontic treatment. The authors found no correlation with surgical occlusion, but rather with the amount of surgical movement. On average, patients had 5.1 ± 2.2 occlusal contacts. Moreover, in over 30% of the cases, occlusal contacts were distributed across less than three out of three segments, which also had no correlation to skeletal stability. In the current study, the minimum requirement regarding number and distribution of occlusal contacts found by Lo et al. [31] was met by both virtual occlusion methods. With over 11 equally distributed occlusal contacts, the required number was doubled by the virtual occlusion, although it was still significantly lower than with the conventional method resulting in over 14 contacts. However, with new concepts of osteosynthesis offering a more stable bony fixation, occlusal stability does not seem to be paramount for skeletal stability [32, 33]. On the contrary, it must be debated whether achieving maximal intercuspation is crucial or even impeding at this juncture of treatment [24, 25, 34–36]. Certain malformations or malocclusions are minimally amenable to orthodontic correction pre-surgically. In these cases, “locked” occlusions must be opened up by surgery before orthodontic movement can commence [24].

Moreover, this study also assessed the time taken for occlusal setting. We found that while the manual method was fastest, virtual methods took a comparable amount of time to complete. The mixed reality method seemed more intuitive, as the pilot cases took less time to complete, however the digital method was faster once the observers were familiar with the spring approach. When overall preparation time was considered, the virtual methods were much faster compared to the conventional method as time-consuming 3D printing was negated. The advantages of virtual occlusion are summarized in Table 1.

We must state that, while the new method of virtual occlusion was highly comparable to the existing one, the current results primarily highlight a more intuitive learning curve as the only objective advantage over the digital method of the IPS Case Designer. However, a limitation of this study is that only orthodontic-first cases with Angle Class II or III malocclusions were included, while anterior open bites and surgery-first cases were excluded. Since setting the surgical occlusion is typically more complex in the latter cases, with the potential need to intentionally lock the bite posteriorly, the semi-automatic approach of the digital method might reach its limits. Hence, we found, similar to Sabev et al., that the spring approach alone did not always lead to satisfactory results, and a combination with manual free movement was needed in some cases. This resulted in a more intricate back-and-forth between the two modes until the desirable occlusion was achieved. In such scenarios, the new mixed reality method, being fully manual and offering the above-mentioned advantage of unrestricted freedom, might prove superior, warranting further investigation in future research which should include clinical studies of actual surgical planning in real cases. Beek et al. carried out a clinical study to evaluate surgical accuracy by comparing conventional surgical occlusion setting using plaster casts versus virtual occlusion using intraoral scans IPS^®^ CaseDesigner (KLS Martin SE & Co. KG, Tuttlingen, Germany) [37]. All surgical procedures were planned with the same software and surgery was performed using CAD/CAM occlusal wafers. The authors found that surgical pitch correction for the maxilla was more accurate using virtual occlusion approach. However, the study did not evaluate the quality of the surgical occlusion. This analysis evaluated the quality of surgical transfer and splint fabrication rather than surgical occlusion. Nevertheless, the authors concluded that virtual occlusion setting is clinically feasible and did not lead to an impaired surgical outcome with regards to maxillary positioning [37]. However, long-term studies evaluating occlusal stability, post-surgical orthodontic treatment and relapse rate are needed to further elucidate the effectiveness of virtual occlusion.

This study showed that the utilization of mixed reality technology shows promising potential for virtual occlusion setting. While it helped to make the manual approach feasible, a combination with automated algorithms like the spring approach might even enhance the procedure. Additionally, integration with a haptic feedback simulation framework, if feasible in the future, could closely replicate the conventional method in a realistic environment, achieving functionality and versatility comparable to the gold standard while retaining the advantages of virtual occlusion.

**Table 1:** Advantages of virtual occlusion compared to conventional occlusion

	Virtual occlusion	Conventional occlusion
Resources	o Elimination of physical models saves time, money and physical storage space	o More labour- and time-consuming to produce physical dental models o Requires a physical storage space
Intra-oral scan and impression-making	o Higher accuracy of intraoral scans o More comfortable than conventional impression-taking	o Impression materials have inherent degree of inaccuracies o Impression-taking in patients with orthodontic brackets results in deformation of the impression material o More labour- and time-intensive to make impressions
Inter-disciplinary management	o Easier to share digital data than physical models o Enables multi-disciplinary virtual meetings with orthodontists, surgeons and engineers	o Physical models have to be shipped to surgeons or orthodontists if they are not in the same facility
Virtual surgical planning	o Allows easy re-setting of occlusion if it is not congruent with the planned skeletal movements o Allows 3D simulation of the surgical outcomes with different surgical occlusions o Allows digital occlusal modifications	o More labour- and time-consuming to reset the surgical occlusion if it is not acceptable o Potential damage to physical models during manual occlusal modificaitions

## Conclusion

The outcomes of the newly developed mixed reality tool for virtual surgical occlusion setting exhibit clear comparability to the conventional method utilizing physical dental models as the benchmark. Maxillary movement values fell within the defined tolerance interval, established as the mean absolute difference between observers in the conventional approach. Notably, deviations at the upper incisal, particularly along the x-axis, remained minimal, rarely exceeding 1 mm, whereas deviations along the z-axis reached a maximum of 1.5 mm. Nevertheless, more than half of all deviations were below 0.5 mm. Consequently, occlusal parameters such as mean midline deviation and overbite remained consistent, while mean overjet increased marginally by 0.32 mm. The number of occlusal contacts, although lower compared to the conventional method (11.2 vs. 14.5), exhibited symmetrical distribution across the dental arch, and surpassed the surgical occlusion requirements for skeletal stability as reported in the literature.

The mixed reality approach, devoid of automatic or semi-automatic calculations inherent in other virtual occlusion tools, relies on manual intervention combined with immersive 3D visualization, affording users a significant degree of flexibility. This characteristic may prove advantageous in specific clinical scenarios, particularly those involving bite situations with vertical discrepancies. Additionally, the learning curve associated with this method was steeper when contrasted with the occlusion wizard of the IPS Case Designer.

Although the clinical applicability of this method is evident, future investigations should delve into long-term considerations, including occlusal stability, compatibility with post-surgical orthodontic interventions and potential effects on recurrence rates. Nevertheless, mixed reality technology demonstrates clear advantages in the realm of orthognathic surgical planning.

## Data Availability

Data is provided within the manuscript.
